# Supplementary material for: Production of Hydrophobic Microparticles at Safe-To-Inject Sizes for Intravascular Administration
Source: Pharmaceutics. 2025 Jan 6;17(1):64. doi: 10.3390/pharmaceutics17010064 (PMC11768317; doi:10.3390/pharmaceutics17010064)
Supplement: Supplementary file 1 [file pharmaceutics-17-00064-s001.zip › pharmaceutics-3398593-supplementary.pdf]

Supplementary Information to

Production of Hydrophobic Microparticles at Safe-To-Inject Sizes for Intravascular Administration

F.L. Gomes, F. Conceição, L. Moreira Teixeira, J. Leijten, and P. Jonkheijm.

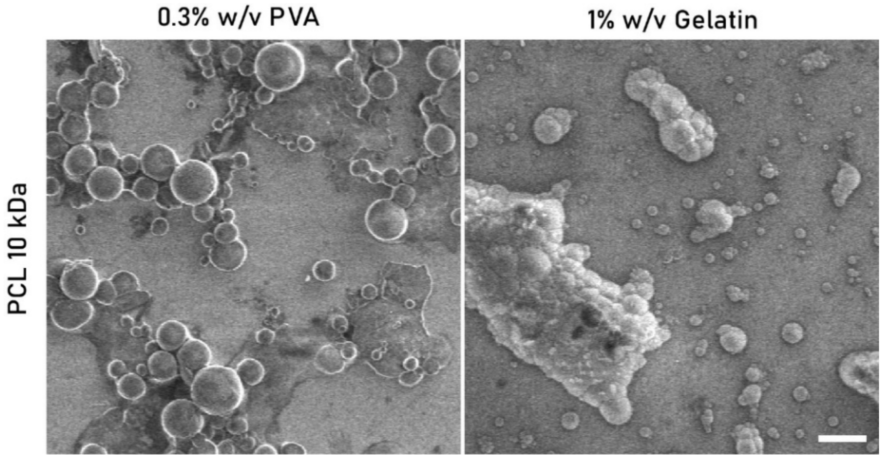

Supplementary Figure S1. Scanning electron micrographs of PCL MPs produced at 10 kDa using PVA or gelatin as stabilizers. Scale bar equals 5 µm.

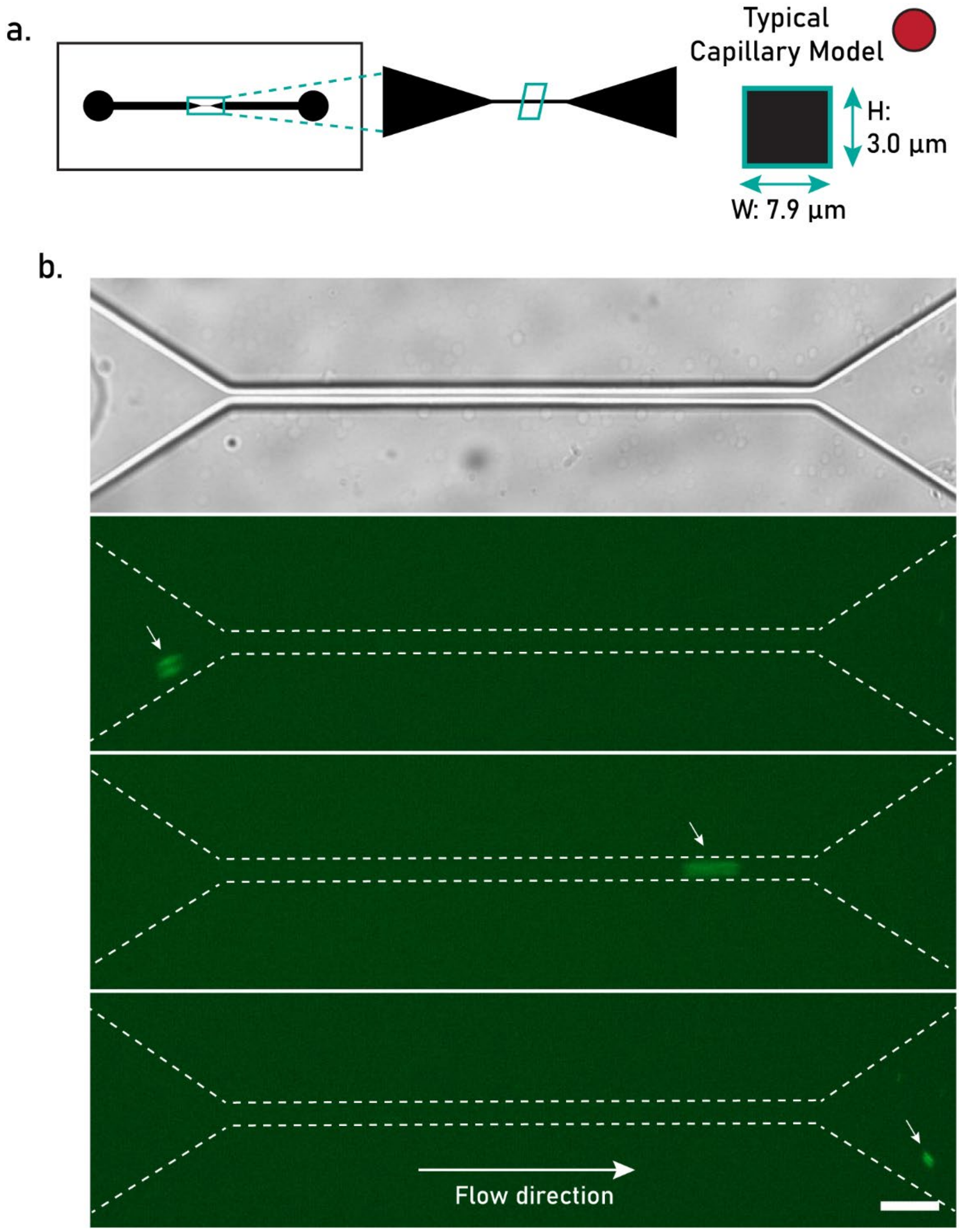

Supplementary Figure S2. a. Schematic depiction of a blood capillary network. **b.** Schematic depiction of chip design for a microfluidics model of a typical (average) blood capillary. **c.** Epifluorescence image of a PCL MP (stained with $DiOC_6$, in green) during flow. Arrows indicate particle. Dashed line marks the channel. Scale bar equals 10 µm.
